# The Conversion of a Peer Teaching Course in the Puncture of Peripheral Veins for Medical Students into an Interprofessional Course

**DOI:** 10.3205/zma001020

**Published:** 2016-04-29

**Authors:** Beate Gabriele Brem, Noemi Schaffner, Claudia Anna Barbara Schlegel, Veronika Fritschi, Kai Philipp Schnabel

**Affiliations:** 1 University of Bern, Institute of Medical Education, Bern, Switzerland; 2 Bern University of Applied Sciences, Department of Healthcare, Bern, Switzerland; 3 Bern Center of Higher Education of Nusing, Bern, Switzerland; 4 University Hospital of Bern, Department of Intensive Care, Bern, Switzerland

**Keywords:** Interprofessional Education, Clinical Skills, Peer Teaching

## Abstract

**Objective: **There is a great interest on both a national and international level in promoting cooperation between different occupational groups within the healthcare professions through interprofessional education (IPE) [1], [2], [3]. Within this project, a peer teaching course on the puncture of peripheral veins was therefore converted from a course for medical students into an IPE learning unit. Students from different occupational groups were to learn within the context of this course, according to the definition from the World Health Organisation (WHO), with and from each other [1].

**Project description: **This course constituted a small group class in the peer teaching format. The didactic principle was based on the idea that the students were to practice the respective practical skills in pairs and give each other reciprocal feedback. Together with the Department for Health at the Bern University of Applied Sciences (BUAS) and the Bern Center of Higher Education of Nusing (BCHEN), the course, which was conducted by the Institute of Medical Education at the University of Bern (IME), was converted into a voluntary IPE pilot project. Students from all three institutions were represented in terms of participants as well as tutors.

**Results: **The course was evaluated very positively by participants, peer tutors and the participating institutions. By means of an OSCE, it could be proven that the course content had been successfully imparted. On the basis of these results, it was determined that the course should be compulsory in the future for students at all three institutions.

**Discussion: **The evaluation results show the successful conversion of the course into an IPE format within the context of the pilot project. The interactive format of the course created the prerequisite that the students from different professional groups learned with and from each other in actuality, and did not just study the same objectives at the same time as with multiprofessional learning. Cooperation between the three institutions is a cornerstone for the development of a research structure which may examine the effect of IPE in the future.

## Zusammenfassung

**Zielsetzung: **National und international besteht ein großes Interesse daran, die Zusammenarbeit der unterschiedlichen Berufsgruppen in den Gesundheitsberufen durch interprofessionelle Ausbildung (IPE) zu fördern [1], [2], [3]. In diesem Projekt wurde deshalb ein Peer Teaching Kurs in Punktion peripherer Venen aus dem Unterricht für Studierende der Humanmedizin in IPE überführt. Studierende unterschiedlicher Berufsgruppen sollen dabei im Rahmen des Unterrichts entsprechend der Definition der Weltgesundheitsorganisation (WHO) mit- und voneinander lernen [1].

**Projektbeschreibung: **Bei dem Kurs handelt es sich um Kleingruppenunterricht im Peer Teaching Format. Das didaktische Prinzip beruht darauf, dass die Studierenden die entsprechenden praktischen Fertigkeiten paarweise üben und sich dabei gegenseitig Feedback geben. Gemeinsam mit dem Fachbereich Gesundheit der Berner Fachhochschule (BFH) und dem Bildungszentrum Pflege (BZ Pflege) Bern wurde der vom Institut für Medizinische Lehre der Universität Bern durchgeführte Kurs im Rahmen eines freiwilligen Pilotprojektes in IPE überführt. Sowohl bei den Teilnehmern als auch bei den Tutoren waren Studierende aller drei Institutionen vertreten.

**Ergebnisse: **Der Kurs wurde von Teilnehmern, Peer Tutoren und beteiligten Institutionen sehr positiv evaluiert. Mit einer OSCE Prüfung konnte nachgewiesen werden, dass die Lehrinhalte des Kurses erfolgreich vermittelt wurden. Aufgrund dieser Ergebnisse soll der Kurs zukünftig verpflichtend für Studierende aller drei Institutionen angeboten werden.

**Diskussion: **Die Evaluationsergebnisse zeigen die gelungene Überführung des Kurses in IPE im Rahmen des Pilotprojektes. Das interaktive Format des Kurses schafft hierbei die Voraussetzungen, dass die Studierenden der unterschiedlichen Berufsgruppen tatsächlich mit- und voneinander lernen und nicht nur wie beim multiprofessionellen Lernen gleichzeitig dasselbe. Die Zusammenarbeit der drei Institutionen ist ein Grundstein für den Aufbau einer Forschungsstruktur, die den Effekt der IPE in der Zukunft beleuchten kann.

## List of abbreviations

- BCHEN = Bern Center of Higher Education of Nusing
- BISS = Bern Interdisciplinary Skills and Simulated Patient Center
- BUAS = Bern University of Applied Sciences, Department for Health
- GMA = Gesellschaft für Medizinische Ausbildung
- IME = Institute of Medical Education, University of Bern
- IPE = Interprofessional Education / Interprofessional Training
- OSCE = Objective Structured Clinical Examination
- PVC = peripheral venous catheter
- SCLO = Swiss Catalogue of Learning Objectives for Undergraduate Training
- WHO = World Health Organization

## 1. Introduction

In their 2010 “Framework for Action on Interprofessional Education and Collaborative Practice,” the World Health Organization (WHO) called for the promotion of interprofessional education (IPE) and practice between healthcare professionals. This call was justified with the following advantages, among others: improved treatment by specialists, better treatment results in cases of chronic illness, reduced complications, greater levels of satisfaction for patients and medical staff, etc. [1]. 

In a position paper from the committee for IPE of the Gesellschaft für Medizinische Ausbildung (GMA), the status of IPE in selected European countries (including Germany, Austria and Switzerland) was summarized. Its status in these countries is very heterogeneous. Scientifically, there are many indications as to the advantages of IPE; however, established findings are still pending [2].

In Switzerland, IPE has been encouraged by political representatives. For example, the promotion of cooperation between different healthcare professions, as well as an adaptation to training and further education courses, was explicitly called for by the Swiss Federal Council in a strategy paper in 2013 [3]. For medical faculties, the role of “collaborator” is codified within CanMed roles in the Swiss Catalogue of Learning Objectives for Undergraduate Training (SCLO) [4]. Accordingly, ever more IPE courses have been offered by the medical faculty at the University of Bern over the last few years. Interprofessional courses about the preparation for a career as a care assistant, an anatomy course [5] and a course on medical confidentiality have already been established as part of this. 

In the following study a project is described in which a peer teaching course on peripheral venipuncture for medical students at the University of Bern (in their 3^rd^ year of study) was converted into an IPE format. According to the literature there are many advantages of students being taught by tutors who have the same status (=peers): Motivated student teachers can replace lecturers who are doctors without the teaching suffering in quality as a quality ([6]; Peer tutors can expand upon their own learned skills and receive insights into didactic concepts [7]; As a result, they learn more and prepare for a future role as a medical tutor [7]; Considering the fact that a doctor must impart knowledge to a patient, the peer tutors also prepare for this aspect of the conversation they will have to carry out as doctors [8].

As regards to IPE, a further advantage of peer tutors which has emerged, is that within the context of IPE, a common problem is that lecturers have been socialized within the context of traditional hierarchical systems [9]. As Baker et al. reported in 2011, some doctors perceive IPE as a threat to their status. Correspondingly, they attempt to reinforce their own interests and professional influence within the context of IPE [10]. To ensure good cooperation within an interprofessional team, a form of leadership based on joint decision making is required, which represents a challenge to lecturers with traditional, hierarchical role models [11]. In contrast to lecturers with years of experience, peer tutors’ role models have yet to be defined [12], and hierarchical gradients between tutors and students are at a minimum [13], [14]. 

In the course, knowledge on taking blood samples and the insertion of a peripheral venous catheter (PVC) was imparted. In practice, these activities can be carried out by the doctor as well as the nursing staff [4]. They are therefore included in the national Swiss Catalogue of Learning Objectives for Undergraduate Training (SCLO) [4] as well as the curricula of the Bachelor degree course for nursing and midwifery at the Bern University of Applied Sciences (BUAS) and the Bern Center of Higher Education of Nusing (BCHEN). 

The course was converted to an IPE format with the following line of questioning within the context of a pilot project:

1. Does a sufficient level of interest exist to convert the existing course from a module at the medical faculty into an interprofessional course?
2. How can organizational problems in relation to administration, course material, etc. be overcome?
3. Is interprofessional peer teaching effective in imparting the content of the course (taking blood samples/inserting a PVC)?

For these purposes, IPE should be understood in the sense of the WHO definition: “Interprofessional Education occurs when two or more professions learn with, from and about each other to enable effective collaboration and improve health outcomes.”[1]. Sotta et al. differentiate interprofessional learning from multiprofessional learning [15]. Thus, interprofessional learning is characterized by interactive learning by which the students can learn with and from each other. In contrast, within the context of multiprofessional learning, participants from different occupational groups study the same objectives simultaneously, but do not learn from each other.

## 2. Project description

### 2.1. Didactic concept

Since 2013, the Institute of Medical Education at the University of Bern (IME) has conducted a course in peripheral venipuncture (taking blood samples and inserting a PVC) for medical students in their 3rd year of study using a peer teaching method. The group size is 4-6 participants and the course is held twice, with each session lasting two hours. 6 medical students were trained initially, who at the time of recruitment were employed at the Bern Interdisciplinary Skills and Simulation Patient Center (BISS). In addition, a specialist (medical staff member at IME or clinical specialist at the Clinic for Intensive Medical at Bern University Hospital) supervises 3-4 small groups on site. 

The teaching concept was composed of the following elements:

- **Observational Learning:** The skill to be learned is demonstrated and then practiced by the participant. This is particularly suited to the learning of simple manual skills [16].
- **Formative Assessment and Feedback: **The participants observe their own as well as their partners` practice using observation sheets. After this, self reflection and reciprocal feedback takes places, since formative assessment and feedback are central elements of successful learning [17].
- **Repetition: **Repetition helps students to consolidate what they have learned and expand upon it [18]. The course was therefore held as two course sessions of two hours each. In the first part, models were practiced upon. Three models per group were available to the students (Standard Venipuncture Arm, Part 00330 Limbs & Things; ACF Pad Venipuncture, Part 00140 Limbs & Things; IV Injection trainer for fastening, item number R16614 Erler Zimmer). In the second part of the course, the learned skill was repeated and once more practiced in a new context (the participants were allowed to take each other’s bloods and/or insert a PVC).

#### 2.2. Planning the interprofessional pilot project

In Autumn of 2013, the IME, BUAS and BCHEN came to the decision to conduct the course interprofessionally. To organize this, representatives from all three institutions held regular meetings. The project managers from the BUAS and BCHEN reviewed the class materials (script, models, etc.) and conducted lesson observations of the tutor training as well as of course sessions. In this phase, no differences between standards at the three institutions were unveiled, other than the fact that hygiene regulations in relation to protective clothing, etc. were administered in a stricter manner by the BUAS and BCHEN. Therefore, scrubs were purchased as protective clothing and the guidelines were adapted for the course.

The biggest problem in planning was the search for a common time slot in which to carry out the course. It was therefore decided that the interprofessional implementation of the course would be firstly tested within the context of a pilot project. Participation was obligatory for medical students, as before. For the participants from the BCHEN and the BUAS, the course was initially offered as an optional course at the respective institutions. In this way, students from both institutions were enabled to participate, even though the time slot for the course partially overlapped with other courses. In 2014, 148 medical students, 11 students from the BUAS and 6 students from the BCHEN participated in the course. In 2015, the numbers increased to 187 medical students, 30 participants from the BUAS and 8 participants from the BCHEN. In the allocation of students of the course, it was ensured that participants from the areas of nursing and midwifery were allocated equally to the individual groups. In 2015, 91 course sessions were carried out (46 sessions for the 1st part and 45 sessions for the 2nd part of the course). At 48 learning sessions, students from 2 institutions attended, and at 3 sessions, students from all 3 institutions attended. At 40 learning sessions it was exclusively medical students who attended, whereby out of these 40 learning sessions, 25 sessions were taught by peer tutors from the BUAS or the BCHEN, respectively. 

#### 2.3. Administration, Tutors, Rooms, Material

The overall coordination of the course was organized by the IME in close conjunction with the BUAS and the BCHEN. 

Students from all three institutions were recruited on a voluntary basis and the teaching hours were allocated evenly to all tutors. In 2014, there were 10 tutors (6 x medicine, 2 x BCHEN and 2 x BUAS). Corresponding to the high participant numbers, in 2015 more tutors were enlisted (5 x medicine, 5 x BCHEN, 4 x BUAS). The tutors were trained by medical staff and a clinical specialist (2 hours of training in technique, 2 hours of training in didactics). Within the context of didactics training, the tutors were taught the 4 step approach according to Peyton [19], because this has been tried and tested for the teaching of simple technical skills [13]. 

Informal solutions were found for the organization of rooms and materials in the pilot project. The peer tutors were paid by their respective institutions. On this occasion, the BUAS and the BCHEN provided more tutors than participants proportionally. In contrast, the rooms, material and administration for the course were provided by the medical faculty. 

#### 2.4. Evaluation of participant satisfaction

For the evaluation of the course, at the end of the 2nd learning session, each small group of 4-6 participants filled out a questionnaire with the following questions:

1. What was good about the course?
2. What could be improved?
3. Was the script helpful? Should anything about it be improved?
4. Other comments?

The students filled in free-text answers on the questionnaires, which were usually formulated in bullet points (i.e. for question 1 What was good about the course? “Small group”). These free-text entries were analyzed with a content analysis according to the principles of a frequency analysis [20]. In this method, quantitative analytical methods such as frequency analysis are applied to a qualitative content analysis [21]. The individual key points of the answers (i.e. for question 1 “small groups”) were viewed as units. Categories were formed based on these units into which equivalent key points were sorted (i.e. for question 1 “group size”). Then it was recorded how often in the questionnaires key points per category were mentioned (i.e. 16 out of 42 groups cited the group size in the questionnaire as something which was to be highlighted as positive).

Additionally, within the context of quality assurance, once per semester a group meeting between delegates from the course program and those responsible for the course in practical skills took place. Within the context of this meeting, oral feedback was collected, which was documented and recorded in a tabular format. Participants were asked about positive aspects and what could be improved. The results were reported back to those responsible for the course respectively or in a private conversation.

#### 2.5. OSCE

The evaluation in relation to the learning outcomes of the course could take place within the context of the pilot project exclusively for medical students, as the course was an additional option for participants from the other institutions to their own course. 

For medical students, the course was, within the context of the pilot project, in the spring semester of the 3rd year of study. In this year of study, the clinical skills taught are examined after the end of the semester with an OSCE, which is also part of the Bachelor examination for the Medicine degree program. The OSCE comprises of 16 stations in total. It was made known to the students that the course content could be included in the exam, but they did not know which stations would actually be examined. An exemplary station for the learning content was developed on blood samples. This station was used in 2014 as one of 16 station for 51 out of 145 students, and in 2015 for 107 out of 185 examination candidates. The checklist on the station was completed within the context of a summative examination by medical lecturers. 

## 3. Results

### 3.1. Participant Feedback

For the evaluation of the course, in Spring Semester of 2015, the small groups filled out a questionnaire on positive feedback and suggestions for improvements together after the completion of the 2nd course session. Such a questionnaire is on hand from 42 out of 45 groups. For 3 groups, the tutors forgot to hand out the questionnaires to the participants. The participant feedback can be summarized as follows: 

It was repeatedly expressed that the class was highly valued. In particular, the participants praised the peer tutors (20 out of 42 questionnaires). Furthermore, the participants liked the small group sizes (16 out of 42 questionnaires), the sequence of the course by which they were able to practice both on a model and each other (13 out of 42 questionnaires), and the fact that there was a sufficient amount of time to practice (9 out of 42 questionnaires). In addition, the good instructions and enjoyable atmosphere of the course were also mentioned. 

In relation to suggestions for improvement, the students noted that it became clear within the course that there were different standards at the individual institutions. This caused uncertainty among the medical students in terms of the upcoming OSCE. Further suggestions for improvements concerned the material and models, rooms and the script. In total, three students expressed the desire for an educational film or an e-learning unit with binding standards. In total, 19 out of 42 groups reported that they saw no need for improvements to the course. A detailed list of the feedback may be found in Table 1 (Tab. 1). 

From the group meeting between delegates from the degree program and those responsible for the course in practical skills, no new additional aspects resulted. The individual responses from the meeting are listed in Table 2 (Tab. 2). 

#### 3.2. Results in the OSCE

Within the context of OSCE examinations, taking blood samples was examined in 2014 and 2015. It was shown that students had learned how to take blood samples according to the expectations of the specialists. Therefore, on average 74±20% (2014) and 82±15% (2015) of all items were correctly carried out by the students which corresponds to the expectations of a successful lesson. For example, in 2014 for the other stations, on average 75±3% of all items were successfully carried out. 

#### 3.3. Feedback from the peer tutors

The responses from the peer tutors on the course were also consistently positive. Many tutors spontaneously expressed the desire to be present for the next implementation of the course. Furthermore, within the context of a debriefing with all tutors in personal conversations and via email, different suggestions for improvements were also collected, on the material employed (advantages and disadvantages of different models), on the script (clearer separation of the instructions on taking blood samples and inserting the PVC) and on tutor training (better standardization of learning content), which will be considered in the further development of the course. 

#### 3.4. Further conversion of the pilot project into an obligatory course for all three institutions

On the basis of the positive responses from the participants and peer tutors, after the course was held in 2015, it was decided that it would be made mandatory for all students at all three institutions. For these purposes, regular meetings with representatives from all three institutions are currently taking place. 

For the institutionalization of the course, it is necessary to solve the issue of the common time slot on a permanent basis. The high level of interest in the course, bolstered by the good evaluation, contributed to the search for a time slot at the participating institutions, which included considering class times on Saturdays or in the evening. In this way, a solution was found at 17:30 in the evening.

While differences regarding standards did not become noticeable during the review of the class materials by representatives from all three institutions nor within the context of the class visitations in the planning phase, these came to light during the implementation of the course. This included details such as whether gloves should be put on at the very beginning or just before the venipuncture. Considering the imminent OSCE, these details were, however, of particular importance to the medical students. To align standards in the future, scripts and treatment instructions from all three institutions, as well as relevant recommendations from literature, will be compiled. Currently, separate treatment analyses for taking blood samples and inserting PVCs are being compiled, which are being viewed and corrected by representatives from all three institutions as required.

At the BUAS and the BCHEN, the learning outcomes of the course will be examined in the future with an OSCE. The use of OSCE stations, which have been a feature of the medical examination up until now and will continue to be in the future, has been proposed. 

In relation to rooms, tutors and materials, the course will be divided proportionally between the three institutions in the future. In this way, it will be guaranteed that comparable conditions will prevail for the lesson at all of the institutions. More models may need to be procured in addition to those already provided. 

## 4. Discussion

Within the context of the featured project, a monoprofessional course in peripheral venous punctuation was converted within the context of a pilot project into an interprofessional course. 

The didactic concept of the course, in which the students worked together in taking blood samples and inserting PVCs, observed each other, and then talked about their experiences using feedback forms, made it possible for students to learn with and from each other. The tutors, who were recruited from all three institutions, also had intensive contact with the 4-6 students in the groups they taught, as after a short theoretical introduction/demonstration (approx. 15-20 out of 110 minutes) they observed them as they practiced, supported them, and gave them additional feedback. Hence, the prerequisite for IPE according to the definition of the WHO [1] and according to the definition by Sotta et al. was met and differentiated from multiprofessional learning [15].

Within the context of the pilot project, it was shown that there was sufficient interest in the interprofessional orientation of the course among students at the BUAS and BCHEN. At no point in time was there a problem in finding student volunteers who wanted to be trained as tutors for the course. There was even an interest in participating in the course, which, considering the fact that the course was additional to their own obligatory courses at their respective institutions and overlapped to some extent with other courses, was not self-explanatory.

 The permanent involvement of the three institutions over the course of the pilot project verifies the interest in conducting the course interprofessionally. This interest, which was reinforced by the positive experiences within the context of the pilot project, led to the further conversion of the course into an obligatory course at all three institutions.

From our point of view, it proved advantageous to firstly conduct a pilot project on a small scale during which the course was made available by the medical faculty to include voluntary participants from the BUAS and the BCHEN. In this way, the course could initially be conducted in the medical faculty’s BISS with the available models, for example. Therefore, it was possible to gain experience in conducting the course interprofessionally, without having to factor in larger investments. Even for organizational problems, such as the search for a common time slot, informal intermediate solutions could be found (i.e. times which did not overlap with other courses for the most part). The successful implementation and positive evaluation of the course bolster, to a great extent, the willingness of all three institutions to carry out the changes as described in Chapter 3.4 (i.e. lesson at off-peak times, procurement of models) for the interprofessional course. They managed, in this way to achieve the prerequisites for the development of IPE programs in overcoming organizational problems, and work according to the recommendation “start small and grow slow” formulated by Burning et. al [22].

The disadvantage of organizing the pilot project with participating students from the BUAS and BCHEN additionally for this optional course lay in the fact that the learning outcomes could not be examined. Learning outcomes for the students from the medical faculty were examined with OSCE stations within the context of their summative Bachelor examination at the end of the 3^rd^ year of study. Within the context of this examination, it could be shown that the course content had been successfully imparted with the example of taking blood samples. Therefore, it may be concluded that interprofessional peer teaching was effective for the participants from the medical faculty at the very least; a further examination for all participants has yet to come and is planned for the future obligatory lesson.

The underlying concept of integrating IPE on the level of peer teaching practical skills is, in our opinion, an excellent opportunity to anchor IPE in the curriculum. We see a high potential to create synergy between the individual institutions, such as an exchange of information between tutors, connecting it to other courses offered, common use of models, etc. Finally, we are yet to prove that our IPE course resulted in a better working relationship between the participating occupational groups or even a better provision of healthcare to patients. This is difficult to prove. In systematic reviews from the Cochrane Database of Systemic Reviews, as well as in multiple updates, we did not succeed in finding a sufficient number of studies to be able to draw generalized conclusions on the effects of IPE [23], [24], [25]. 

For the purposes of proving its use in later practice as promoted in the position paper from the committee for IPE [2], the appropriate research structures must be established. We believe that we have contributed to the creation of such structures with the successful working relationship we developed between our institutions for this course. 

## Acknowledgements

We wish to thank Prof. Theresa Scherer, MME from Bern University of Applied Sciences, Department of Health, for her ongoing involvement in the interprofessional implementation of the course, Norbert Braun from the Institute of Medical Education at the University of Bern for his continuous help in the organization of course material and classrooms, Regula Walter from the dean of studies at the Medical Department, University of Bern for course administration, as well as Dr. Danial Bauer for his helpful proofread of the manuscript.

## Competing interests

The authors declare that they have no competing interests.

## Figures and Tables

**Table 1:**
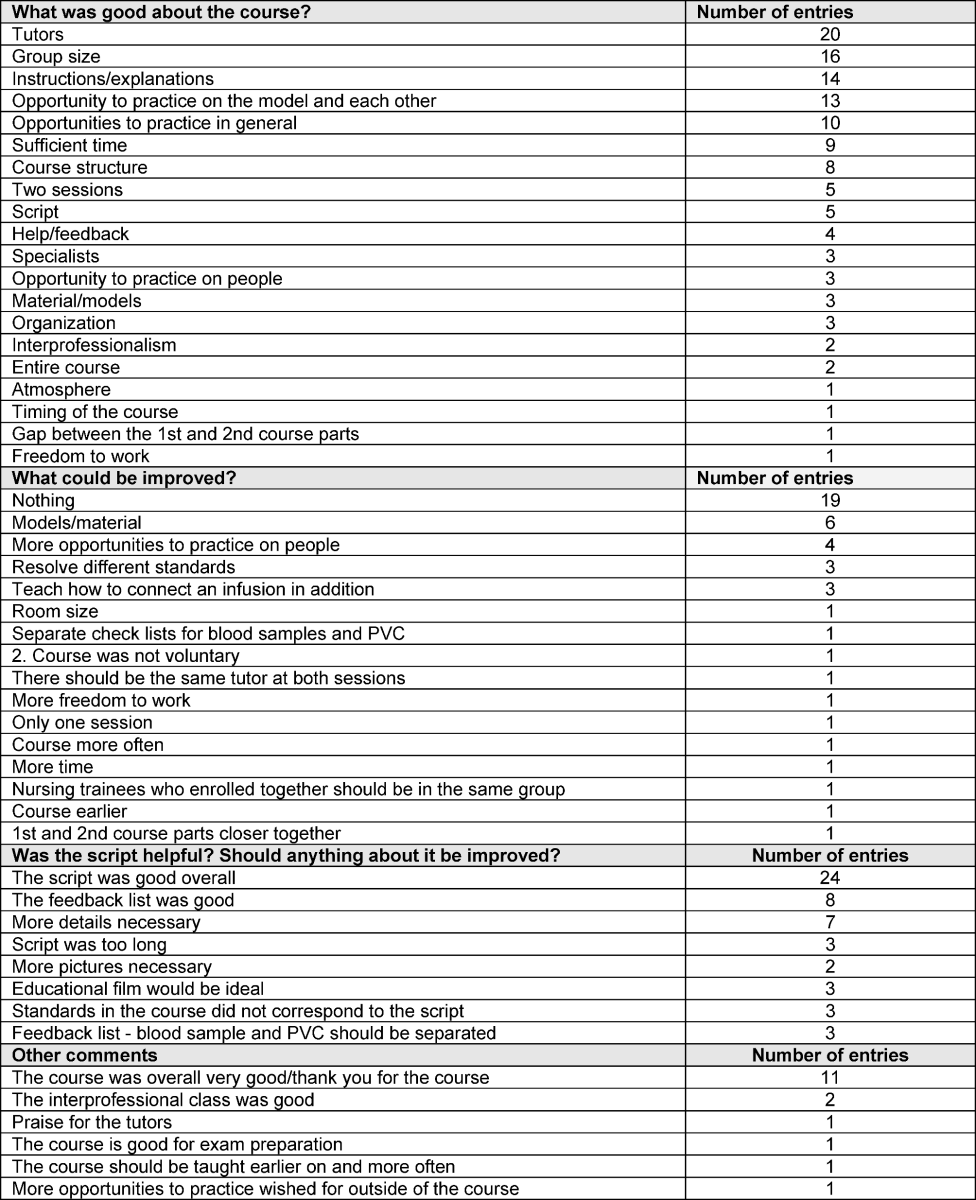
Evaluation of level of participant satisfaction with the course using the questionnaires which were filled out by the course participants (one survey per small group, n=42)

**Table 2:**
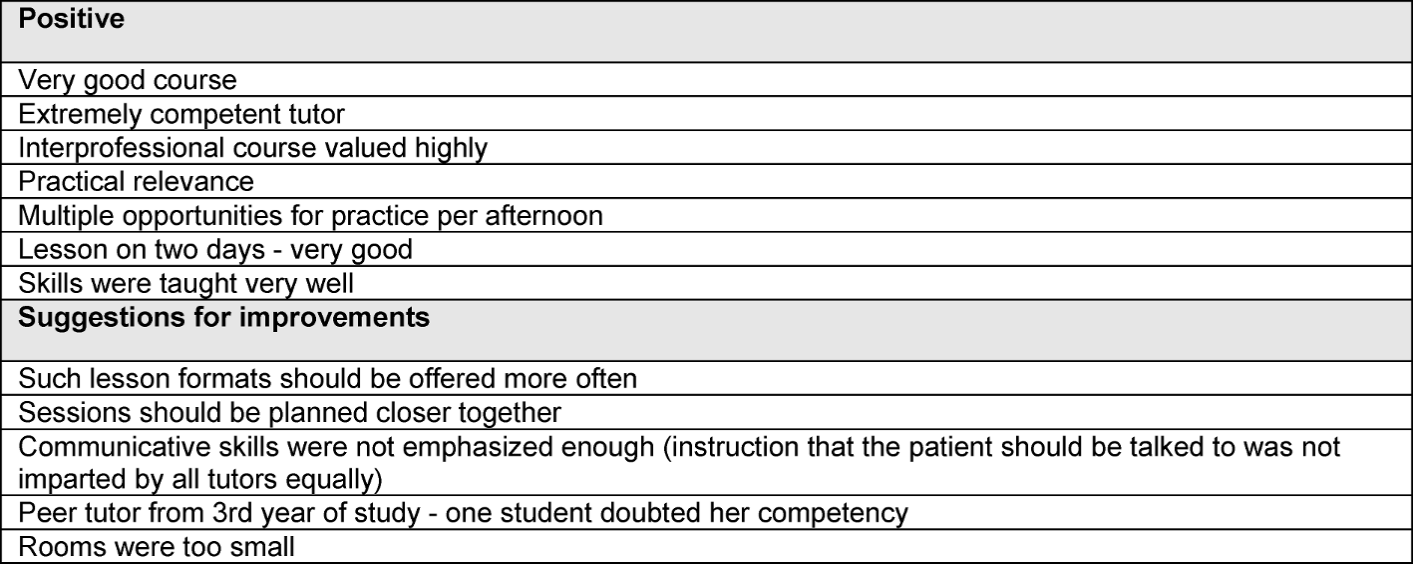
Evaluation of participant satisfaction with the course using responses from delegates from the degree program to those responsible for the lesson in practical skills within the context of a group meeting.
